# On Snake Bites

**Published:** 1854-11

**Authors:** William Hanley

**Affiliations:** Naperville, Ill.


ART. III.—*On Snake Bites.* By WILLIAM HANLEY, M.D., of Naperville, Ill.

July 31st, 7 A.M., was called to H. W. J——ll's, aged 29 years; found him suffering from a severe bite of a rattlesnake, situate on the leg, about an inch above the ankle. The whole limb was swollen as far as the abdomen, and of a purple hue as far as the knee; vomiting a greenish fluid; the surface of the body cold, and bedewed with excessive perspiration; pulse 187, timed by the watch, and respiration very difficult. The wound was inflicted about 3 P.M. on the 30th, whilst walking barefooted in his corn field. He killed the snake, which was a very large one, and possessed fourteen rattles. I was informed that suggested remedies had been employed of the following nature, viz.: giving large portions of brandy, until symptoms of intoxication became manifest, and applying topically spirits of ammonia, snake weed poultice, honey and salt, and indigo, each separate in the order named, without change in his condition, except for the worse. I at once had the limb washed with warm water, and applied spirits terebinth, saturated with nitrate of potassa, freely to the wound, and the entire surface of the limb, employing at the same time considerable friction with both hands. I gave him thirty drops of the same mixture every half hour, in two tablespoonfuls of cold water, until he had taken four doses. The first was swallowed with great difficulty; the second no easier; the third with scarcely any difficulty; and the last quite natural. Vomiting ceased with the first dose; the purple color of the skin began to assume a rose color in about twenty minutes from the first application; and in one hour the limb was much reduced in size. At 9 A.M. I left him, with very little swelling in the limb, an entire disappearance of the purple color of the skin; heat restored to the surface of the body; respiration easy and natural; pulse 85; perspiration reduced to a slight moisture, instead of a complete drenching, as when I first saw him, and a disposition to sleep.

I called at 3 P.M., and found he had slept two hours, and awoke feeling quite well, except a slight head-ache. I was induced to try the above remedy from the following considerations: terebinth being a styptic of the first class, and a rapidly diffusive stimulus, I looked upon it as suitable for employment in this case, for the purpose of restoring heat, and acting as a styptic to the cutaneous circulation. I combined nitrate potassa with the same, in consequence of its well known antiseptic and vivificating influence on the blood.

The *modus operandi* is that of a diffusible and vivificating stimulus, producing an antiseptic condition of the blood, bringing on rapid contraction of the capillaries, adapting the calibres of vessels to the diminished column of blood, (caused by effusion of its serum between the cuticle and tissue,) restoring a healthy cutaneous circulation, and producing a sense of warmth and comfort, instead of coldness and death like exhaustion.

Two other cases of snake bite came under my observation—one August 6th, the other August 10th—the former a young man, aged 18, bit on the arm whilst binding oats; the latter a girl, aged 9 years, bit on the foot whilst playing. Neither of them manifested any other symptom than swelling, and a slight discoloration around the wound. I treated both with the terebinthinate mixture with satisfactory results.

